# Invited Discussion on: Mesh Suspension Thread for Facial Rejuvenation

**DOI:** 10.1007/s00266-020-01670-w

**Published:** 2020-03-24

**Authors:** Woffles T. L. Wu, Bryan Mendelson

**Affiliations:** 1 Camden Medical Centre, 1 Orchard Boulevard. Suite 09-02, Singapore, 248694 Singapore; 2 Centre for Facial Plastic Surgery, 109 Mathoura Rd Toorak.3142, Melbourne, Australia

*Level of Evidence V* This journal requires that authors assign a level of evidence to each article. For a full description of these Evidence-Based Medicine ratings, please refer to the Table of Contents or the online Instructions to Authors www.12springer.com/00266.

This is a small study of a minimally invasive facelifting technique with 21 responders, of the short- to mid-term effects after a specially designed facial lifting polypropylene thread called the mesh suspension thread that is inserted into the facial soft tissues via a trocar and cannula, introduced from the temporal area down to the distal face. The cannula is then removed leaving the threads in situ, which are subsequently secured proximally to the deep temporal fascia. This unique thread features a mesh-like configuration at its distal end and some barbs in the proximal end.

All subjects were followed up and assessed by the patients themselves as well as an independent rater for between 6 and 24 months using the Global Aesthetic Improvement Scale (GAIS), the Modified Fitzpatrick Wrinkle Scale and the Marionette Lines Grading Scale (MLGS). The authors observed that improved results could be maintained up to one year (in 14 patients) and up to two years (in 8 patients). They also noted that in some cases the independent rater gave a lower score than the patients who gave themselves and who remained satisfied with their own results. Their conclusion was that this novel thread could provide a long-lasting result, up to two years. Three sets of pre-operative and follow-up photographs were used to illustrate this point.

They then embark on a discussion based on the literature cited in their references, which examines the merits of traditional facelifting versus the various threadlifting techniques available and the history of barbed threads in our specialty.

The study is for the most part neatly conducted and summarised, for which the authors are to be commended. The technique and the mesh thread used are also conceptually interesting as they introduce yet another way to increase the longevity of these types of minimally invasive sutures. The sample size of 21 responders is not insignificant, and some useful information can be gleaned from this.

We have, however, some issues with the interpretation of the results, the photographs used and the lack of references cited on the history of barbed threads. Had they been correctly sourced, the narrative of the discussion may have changed. As it is, the discussion is somewhat skewed.

First, the photographs are inconsistent in the basics of both lighting and head positioning. Some were taken in shadow, but others not. Furthermore, only three patients (patients 2, 11 and 16) out of 21 responders were shown. It would have been more useful if the authors had illustrated all eight cases they deemed to have good results at 2 years since the long-term results are the thrust of their paper.

In evaluating pre- and post-op results in any traditional facelift or threadlift case, it is important to have good ¾ oblique views which show clearly the Ogee line of the far-side face as well as the jawline and jowls of the near side. It is these two lines or curves that most clearly convey the youthfulness or maturity of the face. The Ogee line allows us to better assess the elevation of the cheeks or jowls and any narrowing of the lower face/jowl area. In this paper, the oblique photographs of patients 2 and 11 are turned away too laterally for this, such that either the nose or the lips obscure full assessment of the Ogee curve.

Patient 16 (Fig. 3) has good before and after ¾ oblique views of the far-side Ogee curve and near-side jawline, but unfortunately no real difference is seen between the pre-operative and 2-year two-month post-operative pictures. It is only on the AP view that patient 16 shows some improvement of the marionette line and jowl configuration at 2 years and 2 months. In the other two cases illustrated, both on AP and ¾ oblique views, it is difficult to discern any improvement at 1 week or 2 years post-operatively. To summarise, based on the three cases illustrated (one of whom shows a slight improvement only on the AP view), it is difficult to accept their conclusion that ‘this novel thread could provide a long-lasting result up to two years’.

The authors also stated that the only complications encountered were some pain at the temporal area, tension lines along the cheek and minor irregularities. Presumably, there were no serious complications such as infections or granulomata due to the experience of the surgeons involved, their competent handling of the threads and their constant maintenance of a sterile field during the procedure.

The hallmarks of a good procedure are predictability, reproducibility and reversibility. There must be an easy way out if something goes wrong. Perhaps, this is why the industry has gravitated towards absorbable threads—an avoidance of responsibility when results are less than desirable. After all, the threads will eventually dissolve.

**Fig. 1:**
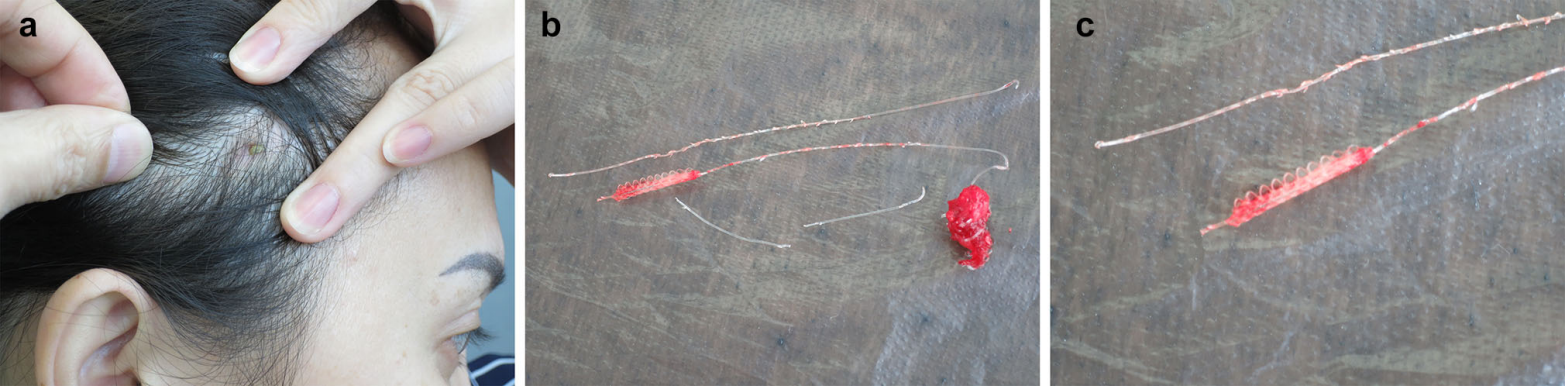
**a** Abscess of the temple following a mesh lift. **b** Removal of infected mesh thread and temporal mesh. **c** Close-up of the mesh portion of the thread

The senior author of this commentary has removed the same type of mesh threads as described in the study from a patient who had developed an abscess over the face and temple after a mesh-lifting procedure (Fig. 1a). The threads proved difficult to remove due to tissue ingrowth into the distal mesh-like nest of the threads which had formed fusiform lumps in the cheeks. Via an incision in the temple, the temporal mesh was first removed and then the infected threads that had developed a column of coagulative necrosis around them were extricated, together with some clumps of necrotic tissue (Fig. 1b, c).

It is a constant concern when these minimally invasive facelifting procedures are touted as ‘lunchtime facelifts’ as this severely dumbs down the procedure. Doctors who are not surgically trained may think these procedures require only minimal sterility, as in the administration of botulinum toxin or facial fillers. Practitioners have been observed performing similar threadlifting procedures using only basic alcohol swabs to prep the face, without sterile drapes and without changing into OT garb. It is no wonder that infections and granulomata are seen in the threadlift world.

In their discussion and the references cited, the authors have surprisingly, completely omitted any papers, commentaries or book chapters on the subject of threadlifting written by Wu, the senior author of this commentary [2–8]. They correctly cite and credit Sulamanidze as the first person to describe the clinical application of barbed threads in the face with his APTOS sutures, but incorrectly list Isse as the next inventor of a facelifting barbed thread and have jumbled up the history of barbed threads somewhat. They also state that to date there are no threadlifting techniques that provide long-term results. This is also incorrect.

Isse in fact learned the technique from and was introduced to the manufacturer of the threads by Wu, after he saw the latter’s presentation at a scientific meeting in Australia in 2003 [9]. This is well documented in a scholarly book chapter on the history of barbed threads by Kress (2008) [10].

**Fig. 2:**
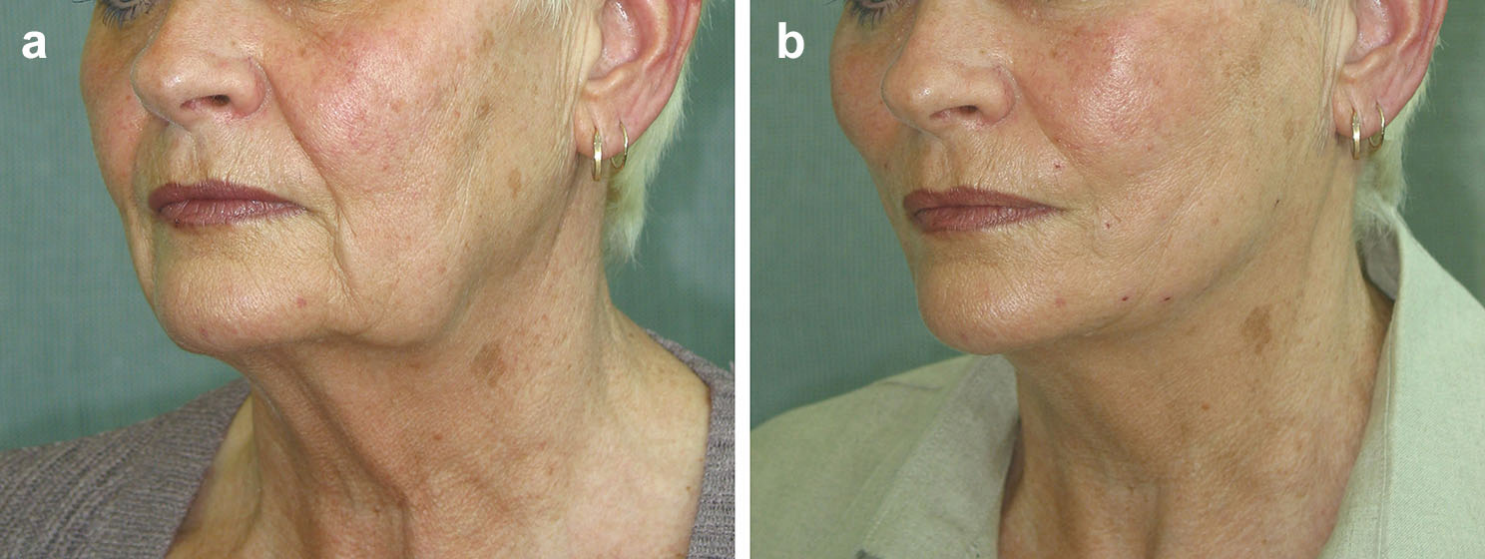
**a** Pre-operative view of a 63-year-old Caucasian woman who wished to have a non-surgical facelifting (2004). **b** Four-day post-operative appearance after the Woffles Lift (eight threads on each side) showing accentuated Ogee curve and restitution of youthful contours with elevated cheekbones, increased malar volume, effacement of nasolabial and marionette lines and improved jawline

After Sulamanidze in 2000, Wu in 2002 devised the first long facelifting barbed thread to create a true facelift effect (Fig. 2a, b). This procedure, named the Woffles Lift, was published in 2004 [2] and has subsequently been described at many international meetings [11–19]. It is understandable that the authors of this present study may be unfamiliar with the use of the Woffles Lift as the Woffles Thread used in the technique is currently not commercially available, but the numerous references to the thread in the literature could have been referred to as stated above.

The Woffles Lift has been used primarily by Wu for the last 18 years and by surgeons who have learned the technique such as Mendelson, the second author of this commentary. As a craniofacial surgeon dedicated to long-term outcomes and the safety of procedures, Wu has continued to observe the effects of the Woffles Lift barbed threads in the face and their low complication rate of 1–2% over this long period of time. The Woffles Threads have been shown to provide long-term support to sagging soft tissues. The threads are easy to remove with a sharp tug should they be inappropriately placed. Sasaki in an independent study found that of all the barbed or cone threads available at the time, the Woffles Thread possessed the greatest lifting capacity with the highest pull-out tension [20].

By way of historical context, Wu, Isse and Ruff were brought together for a special symposium on the use of barbed threads in plastic surgery at the 2006 California Society of Plastic Surgeons Meeting, held in Indian Wells [15]. At this meeting, Wu described his 4-year experience with the Woffles Lift, showing long-term results beyond 2 years, whilst Isse described his 18 months of experience with the barbed threads used in his Isse endolift technique. Ruff discussed the applications of barbed sutures for repairing tendons and for knotless wound closure. It was after this meeting that the suture company Surgical Specialities and Quill merged to form the Contour Thread Company which resulted in the immense popularity of threadlifts.

**Fig. 3:**
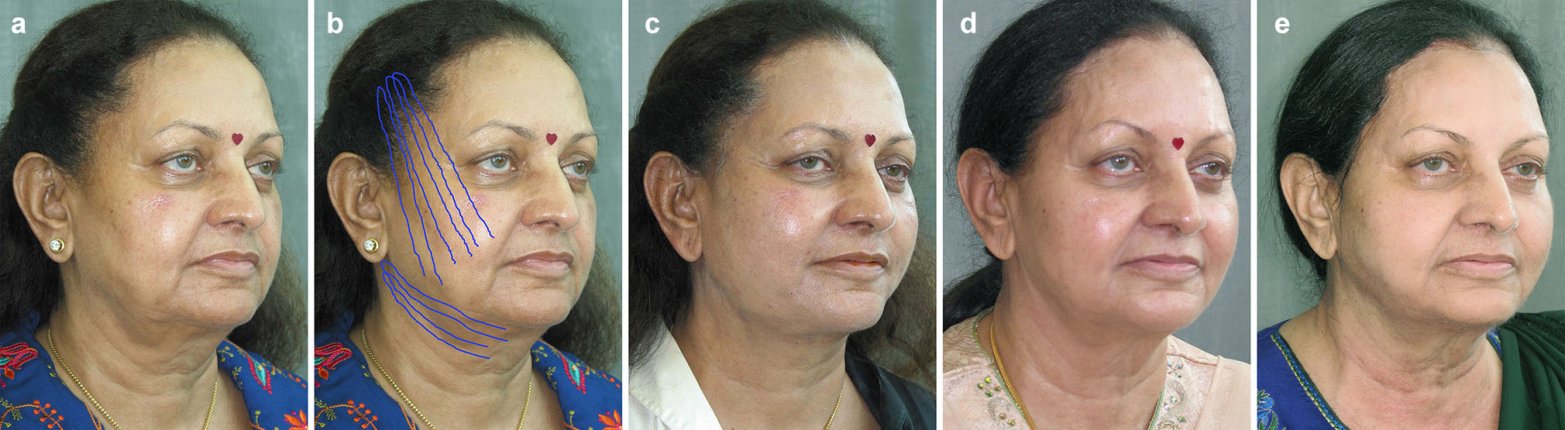
**a** Pre-operative view of a 65 year old Indian woman seeking a minimally invasive facial and neck rejuvenation (2005). **b** The position and vectors of the Woffles threads used in the face and neck are illustrated. **c** 1 week postoperative appearance after Woffles Lift (8 threads on each side of the face, 4 threads on each side of the neck). **d** 5 year follow up (2010). The patient is 70 years old. **e** 7 year follow up (2012) after addition of 4 threads per side in 2011. The patient is now 72 years old

**Fig. 4:**
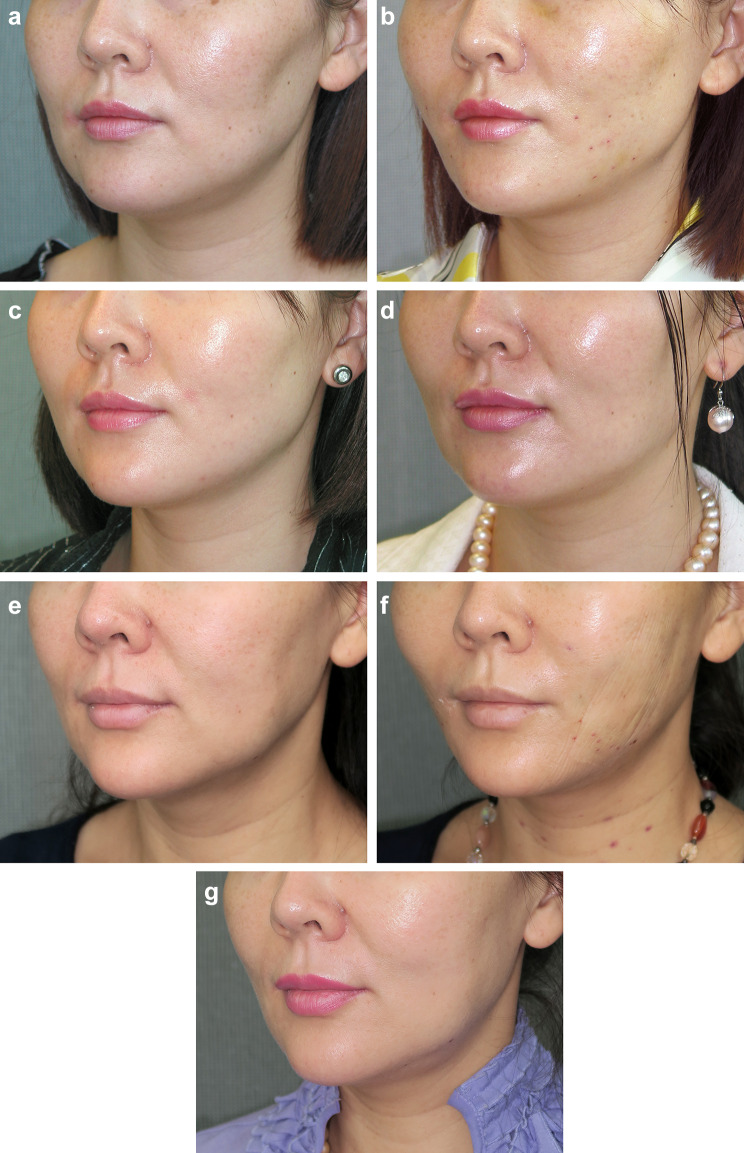
**a** Pre-operative view of a 45-year-old female patient who wanted to improve early jowl and cheek descent with more youthful contours of her face and improvement of the subzygomatic hollows. **b** Immediate post-op, 4 days after the Woffles Lift of the face (eight threads per side)—the cheek is lifted, jowls are diminished and lower face is narrower with good Ogee contouring. **c** Four months post-operative—good contours and shape of face. **d** One year post-operative—the correction of jowls. Midface, and cheek benefit remains evident. **e** Age 54, 9 years after the first Woffles Lift and before the second Woffles Lift—despite 9 years having elapsed the face still looks lifted and better than her pre-operative appearance 9 years previously. **f** Immediate post-op, 3 days after the second Woffles Lift with eight Woffles Threads on each side. The procedure was essentially repeated, without removing any of the original threads—accentuated Ogee curves and correction of ageing deformities seen. **g** Age 57, 2 years after the second Woffles Lift, with good maintenance of a youthful facial shape and appropriate tautness

In recent years, Wu has published the long-term results of his threads at 5 years [5, 6] (Fig. 3a–d) and in one case even 9 years post-operatively [7] (Fig. 4a–g).

Threadlifting procedures have, for the last 20 years, fascinated plastic surgeons and aesthetic physicians around the world because they promised visible facial rejuvenation with minimally invasive techniques and a shorter recovery time. It was then and remains an attractive concept. Whether this promise is fulfilled, only time will tell.

Many different products from barbed sutures to cone threads and now to a polypropylene mesh thread being reviewed have been studied and used clinically with varying results and outcomes. Different materials from non-absorbable polypropylene threads to silicone threads to absorbable PDO threads to hybrid combinations of polypropylene threads mixed with dissolvable cones and even dissolvable PDO threads with dissolvable cones have all been utilised.

In a recent paper, Bertossi et al. [21] studied 160 patients who underwent a threadlift procedure, using barbed PDO threads, and concluded that post-operatively at 1 month there was visible facial lifting, by 6 months this effect had significantly reduced and by 1 year all effect was lost. This correlates well with the findings of Yoon et al. [22] who studied the tissue changes over time after insertion of PDO threads into a pig experimental model and observed that the threads retained their shape for 12 weeks, became fragmented by 24 weeks and completely dissolved by 48 weeks, thus explaining the loss of effect at 1 year of Bertossi’s study.

Most doctors well versed in threadlifting techniques will agree that polypropylene barbed threads or even as in this case, a polypropylene meshed thread, will provide the longest term results. It is perplexing why PDO threads continue to be used when the results are extremely short-lived.

Our apologies to the authors of this paper if we have been too harsh in our criticisms. We still respect and salute you for your well-thought-out study, the carefully documented follow-ups and assessments and your contribution to the ever-expanding literature on threadlifting techniques.
